# Different Statistical Approaches to Investigate Porcine Muscle Metabolome Profiles to Highlight New Biomarkers for Pork Quality Assessment

**DOI:** 10.1371/journal.pone.0149758

**Published:** 2016-02-26

**Authors:** Julia Welzenbach, Christiane Neuhoff, Christian Looft, Karl Schellander, Ernst Tholen, Christine Große-Brinkhaus

**Affiliations:** Institute of Animal Science, University of Bonn, Endenicher Allee 15, 53115 Bonn, Germany; University of Bologna, ITALY

## Abstract

The aim of this study was to elucidate the underlying biochemical processes to identify potential key molecules of meat quality traits drip loss, pH of meat 1 h post-mortem (pH1), pH in meat 24 h post-mortem (pH24) and meat color. An untargeted metabolomics approach detected the profiles of 393 annotated and 1,600 unknown metabolites in 97 Duroc × Pietrain pigs. Despite obvious differences regarding the statistical approaches, the four applied methods, namely correlation analysis, principal component analysis, weighted network analysis (WNA) and random forest regression (RFR), revealed mainly concordant results. Our findings lead to the conclusion that meat quality traits pH1, pH24 and color are strongly influenced by processes of post-mortem energy metabolism like glycolysis and pentose phosphate pathway, whereas drip loss is significantly associated with metabolites of lipid metabolism. In case of drip loss, RFR was the most suitable method to identify reliable biomarkers and to predict the phenotype based on metabolites. On the other hand, WNA provides the best parameters to investigate the metabolite interactions and to clarify the complex molecular background of meat quality traits. In summary, it was possible to attain findings on the interaction of meat quality traits and their underlying biochemical processes. The detected key metabolites might be better indicators of meat quality especially of drip loss than the measured phenotype itself and potentially might be used as bio indicators.

## Introduction

Sensory and technological quality characteristics of meat products are essential for acceptance of consumers and manufacturing industries. The variability of meat quality is high and the regulation of muscle properties influencing meat quality traits is still unclear [1]. One important commercially interesting meat quality parameter is the ability of meat to retain water also known as water-holding capacity (WHC). In order to characterize WHC in pork, drip loss is measured. High drip loss leads to significant reduction of meat quality resulting in monetary losses and reduced acceptance of consumers and meat-processing companies. Regularly drip loss in *Musculus longissimus dorsi* (LD) is around 1 to 5% [2]. Drip loss is affected significantly by the structure of the muscle and the muscle cell itself and by unfavorable slaughtering conditions. Drip loss in particular is influenced negatively by too short rest periods and stress before slaughter that is associated with the rate and extent of muscular pH decline [3]. Furthermore, meat quality attributes are controlled by genetic effects as well, although the heritability for some traits is low. Genetic studies revealed several quantitative trait loci and candidate genes. However, the underlying mechanisms leading to the variation in all meat quality traits need to be better understood [4–6].

Some studies suggest that the levels of metabolites are helpful in order to understand the complex biological mechanisms of the underlying meat quality traits [7]. In this regard, metabolomics is a useful technique to identify candidate biomarkers that influence and indicate complex traits [8], improve preventive health care and enable early recognition of diseases [9]. In animal breeding biomarkers might be used for prediction of economical attractive phenotypes. For example Te Pas et al. [10] and Rohart et al. [11] investigated the suitability of metabolite profiles in prediction of meat quality traits in pigs. Furthermore, investigating metabolites as new phenotypes might allow uncovering the biochemical processes leading to aberrant meat quality. In general, metabolites are closer to the target phenotype compared to the level of the transcriptome or genome. In a current study, Muroya et al. [12] used this characteristic of metabolites to reveal metabolic pathways in different porcine muscle types.

In order to identify reliable metabolite biomarkers and metabolic pathways, eligible approaches of metabolite quantification and annotation are needed. A promising procedure is the untargeted metabolite profiling using mass spectrometry and subsequent data base query. In this situation, caused by the possibility of quantitative high‐throughput analysis of biological samples, the number of measured metabolites is usually much larger than the number of available biological samples. This case is also known as the “large p, small n” problem or rather overfitting [13]. Several methods have been described that are able to handle data sets with a large number of variables [14, 15].

Therefore, the main objective of this study was to analyze the relationships between muscle metabolite profiles and meat quality traits through an untargeted metabolomic approach in order to predict their potential as biomarker and to investigate the underlying molecular structures and processes of meat quality. In regard to the “large p, small n” problem, four different statistical methods, namely correlation analysis, principal component analysis (PCA), random forest regression (RFR) and weighted network analysis (WNA), were applied. Whereas correlation analysis and PCA are appropriate and commonly used methods to investigate the relationship between different variables, RFR and WNA hold several advantages in the analysis of highly multivariate, complex data. The construction of biological networks based on metabolites allows the identification of molecular interactions because they do not only quantify the correlations between pairs of metabolites, but also the extent to which these molecules are connected with other expressed metabolites.

## Material and Methods

### Animals, tissue collection, phenotyping

This study is based on 97 performance-tested F_2_ animals of a reciprocal crossbreed Duroc × Pietrain (Du × Pi). The animals were selected within F_2_ family and based on their extreme high or low values of drip loss. The animals were kept and performance tested under standardized conditions at the Frankenforst experimental farm of the University of Bonn from 2002 until 2007. Data recording and sample collection were conducted strictly in line with the German law on animal welfare. The entire experiment, including applied standard operating procedures, was approved by the veterinary and food inspection, Siegburg, Germany (No. 39600305-547/15). All animals were slaughtered at an average of 180.5 days and average carcass weight of 86.5 kg. The phenotypes were recorded in a commercial slaughterhouse according to the rules of German performance stations [16]. Further information can be found in Liu et al. [4].

In brief, sample collection was performed thoroughly after exsanguination. About 10 minutes post-mortem (p.m.) tissue samples were rapidly dissected, snap-frozen in liquid nitrogen and stored at– 80°C. For further examination we choose the meat quality traits drip loss, meat color, pH in meat 1 h p.m. (pH1) and pH in meat 24 h p.m. (pH24) in LD. Drip loss was measured using the bag method of Honikel and Kim [17]. The samples from LD between 13th/14th rib were collected 24 h p.m., weighed, and wrapped in a plastic bag. After storage for 48 h at 4°C, the samples were reweighed and drip loss were calculated as a percentage of weight loss based on the initial weight of a sample. Muscle color was measured at 24 h p.m. by Opto-Star (Matthaeus, Klausa, Germany). Opto-Star measures the light reflection of the meat and gives it as meat color value. High light reflectance factor stands for pale meat; low reflectance describes dark red meat color. The traits pH1 and pH24 were measured 1 and 24 h p.m. in LD. To describe the relationship between meat quality traits we performed a phenotypic correlation analysis.

### Metabolite profiling

The samples metabolite spectra in LD were measured by Metabolomic Discoveries GmbH (Potsdam, Germany; www.metabolomicdiscoveries.com) via gas chromatography—mass spectrometry (GC-MS) and liquid chromatography—quadrupole time of flight—mass spectrometry (LC-QTOF/MS).

For metabolite extraction frozen muscle tissue was mechanically disrupted in a ball mill in liquid nitrogen. 40 mg of homogenate was mixed with 500 μl 80% (v/v) methanol and incubated for 15 minutes in a thermo shaker (1000 rpm) at 70°C. Cellular debris was removed by centrifugation. 10 μl of the extract were dried and subsequently used for the analysis on GC-MS. For LC-MS 1 μl was injected. Derivatisation and analyses of metabolites by a GC-MS 7890A mass spectrometer (Agilent, Santa Clara, USA) were carried out as described [18]. The LC separation was performed using hydrophilic interaction chromatography with a ZIC-HILIC 3.5 μm, 200 A column (Merck Sequant, Umeå Sweden), operated by an Agilent 1290 UPLC system (Agilent, Santa Clara, USA). The LC mobile phase was a linear gradient from 90% to 70% acetonitrile over 15 min, followed by linear gradient from 70% to 10% acetonitrile over 1 min, 3 min washed with 10% acetonitrile and 3 min reequilibration with 90% acetonitrile. The flow rate was 400 μl/min. Hyphenated mass spectrometry was performed using a 6540 QTOF/MS Detector (Agilent, Santa Clara, USA). The measured metabolite concentration was normalized to the internal standard.

GC-MS and LC-QTOF/MS are used for untargeted metabolite profiling and facilitate the identification and robust quantification (accurate molar mass) of a few hundred metabolites in a single tissue sample. Chromatography followed by mass spectrometry has a relatively broad coverage of compound classes, including organic and amino acids, sugars, sugar alcohols, phosphorylated intermediates and lipophilic compounds. With the combination of both methods it is possible to detect metabolites in a range of 50–1700 Dalton, with a precision of 1–2 ppm and a solution of mass/Δmass = 40.000 (Report METABOLOMIC DISCOVERIES GmbH). For details on the methods see Lisec et al. [18]. Metabolites were identified and annotated in comparison to Metabolomic Discoveries' databases, which resort to Human Metabolome Database (HMDB, www.hmdb.ca), METLIN (www.metlin.scripps.edu/) Lipid Maps (www.lipidmaps.org/). Annotation of metabolites was based on mass assignment, retention behavior and structure information. Not annotated metabolites are characterized by their accurate mass and retention time. The data set of the meat quality parameters and all quantified metabolites is presented in S1 Table.

### Statistical analysis

#### Processing/correction of phenotype and metabolite data

Individual phenotypes of meat quality traits and metabolite expression levels were corrected for systematic effects using a fixed, generalized linear model of R software (www.r-project.org). The linear model contained besides population average *μ* and random residuum *e*, the effect ‘season’ (*S*, 3-month classes) and ‘slaughter weight’ (*SW*) as a linear covariable.

$$Y_{ij}=\;\mu +b\left({SW}_{ij}\right)+\;S_{i}+\;e_{ij}$$ (1)

All further statistical analysis methods were carried out using the calculated residuals of metabolite expression intensities and meat quality characteristics.

#### Association between metabolite profiles and meat quality traits

To investigate associations between metabolite profiles and meat quality traits we applied four different statistical approaches: 1) Correlation analysis, 2) PCA, 3) WNA, 4) RFR. These methods have different properties in order to handle the specific statistical problems (‘large p, small n’, high dimensionality and distinct correlation between variables) of the metabolomics data set. All statistical methods of analysis were performed with R (http://www.r-project.org).

#### Correlation analysis

In a first step simple Pearson correlation coefficients were estimated to investigate the relationship between paired samples of metabolites and meat quality traits. Significant correlations (p ≤ 0.05) were considered for further biological interpretations.

#### Principal Component Analysis (PCA)

The PCA procedure is an unsupervised method which condenses the large number of metabolites into a set of representative, uncorrelated principle components (PCs) by means of their variance covariance structure [19]. Only PCs which explain more than 1.5% of the entire metabolite expression variance were considered for further analysis. The relevance of each metabolite within each PC was quantified by their corresponding loadings.

#### Weighted Network Analysis (WNA)

Similar to the PCA, the WNA procedure [15] tries to reduce the dimensionality of the metabolic information. Simple network statistics were used to generate a limited number of biological interpretable modules. Pearson correlation matrix (adjacency matrix) of all bivariate metabolite comparisons is used to calculate the distances between the metabolites, corresponding to the differential metabolite expression. By raising the absolute value of the Pearson correlation to a power β ≥ 1 (soft thresholding), the weighted network construction emphasizes large correlations at the expense of low correlations [20]. The distances between metabolites are integrated into a topological overlap matrix (TOM) which is used to cluster the variable expression profiles hierarchically. The results are visualized by a dendrogram with hierarchically arranged branches and connected nodes. The branch position of each metabolite indicates the actual connectivity (topology) in the network. The metabolite located at the end of the branch is the most connected nodes (‘hubs’), which play an important role in influencing the co-regulation patterns of other nodes in the network. Moreover these hubs may act as linking nodes for communication and interaction between different networks [21].

For further evaluation the branches were clustered into separate co-regulated modules, which are visualized by different colors in the corresponding dendrogram. The mathematical delimitation of each module was obtained through semi-automated, adaptive pruning of the hierarchical clustering dendrogram. Based on the distance matrix of all metabolites (dissimilarity of TOM) and the hierarchical clustering dendrogram the function produces a vector of numerical labels giving assignment of objects to modules [15].

In a next step the metabolite expression profiles for each module are decomposed via a singular value decomposition to form module eigenvalues (MEs). This procedure is closely linked to a PCA within a module, where the MEs resemble the first PC. The importance of each metabolite for its module (Module membership, MM) is quantified by the correlation between MEs and metabolite expression profiles. Moreover the significance of each module specific metabolite (Metabolite significance, MS) for the response traits is expressed by the Pearson correlation coefficient. The MS values correspond to the Pearson correlation coefficients between metabolites and meat quality traits.

Another method to classify metabolites as key indicators of a metabolic network is the concept of maximum adjacency ratio (MAR). MAR is a function of connectivity that is calculated across all metabolites. Thereby, MAR describes the relativeness of the entire metabolite network. In coexpression networks, MAR is a useful parameter since it allows to determine whether a node forms moderate relationships with a lot of features (MAR_i_ < 0.5) or very strong relationships with relative few features (MAR_i_ > 0.5) [22]. From the viewpoint of network analysis MAR differs from MM because is not a module based parameter, but is able to indicate strong linked-up metabolites, that are involved in many metabolic pathways [23, 24].

The WNA procedure used in our study is implemented in the package ‘Weighted Gene Co-expression Network Analysis’ *(WGCNA)* in R [15]. As an optional feature of *WGCNA*, the user is allowed to assess the minimum number of metabolites contained in each module. To construct an interpretable number of modules, we used the standard thresholding parameter (β) and a minimum of 30 metabolites per module in our analysis [24]. The mathematical delimitation of each module was obtained through pruning of the hierarchical clustering dendrogram implemented in the function ‘cutreedynamic’ of *WGCNA*.

#### Random Forest Regression (RFR)

RFR is a supervised learning tool that estimates the associations between metabolites and response variables (meat quality traits) using tree-based methods with integrated permutation tests [25]. As it has been shown by Strobl et al. [26] and Nicodemus et al. [27], the random forest algorithm is believed to successfully identify relevant predictor metabolites even in high dimensional settings involving complex interaction structures and highly correlated variables. The bootstrapping algorithm implemented in RFR involves two layers of random sampling: response values and metabolite profiles. The RFR procedure is described in detail in Breiman [14] (cf. Text A in S1 File).

Because of important pitfalls of the traditional RFR algorithm by Breiman [14] implemented in R package *randomForest* [23], in this study the RFR routine was calculated based on an alternative class of decision trees developed by Hothorn et al. [24] and Strobl et al. [25]. In the enhanced RFR procedure [26], tree construction and variable importance (VI) estimation is addressed through the principle of non-parametric conditional hypothesis testing (cf. Text B in S1 File).

Essentially, the conditional RFR has the following advantages: the procedure uses the ‘conditional inference forest’ (CIF) methodology as splitting criterion. At each splitting node, each predictor is globally tested for its association with the trait of interest and a p-value is computed. Hence, CIF splitting is based on an essentially unbiased splitting criterion that automatically adjusts for different marginal distributions of the predictors and thus does not share the pitfall of Breiman´s RFR. Moreover the resampling scheme in conditional RFR based on subsampling instead of bootstrap sampling and Strobl et al. [25] recommend to systematically using sampling without replacement to prevent biases in VI measurement. Finally the aggregation procedure in CIFs works by averaging the observation weights extracted from each of the trees and not by averaging predictions directly (majority voting). As a result, even in case of high correlated predictors or variables with wide scale of measurement, modifications of the standard RFR procedure lead to less biased forest construction and VI calculation.

VI calculation based on the permutation principle of ‘mean decrease in accuracy’ (MDA). The so called ‘MDA importance’ or ‘permutation importance’ is directly based on the prediction accuracy rather than on the splitting criterion (see Gini importance [14]). The MDA importance describes the difference between OOB error after random permutation of the relevant predictor where the OOB error results from validation of the original tree. Substitution of a considerable predictor is expected to decrease the OOB error. Therewith high MDA values indicate metabolites with distinct effect on the observed trait. The MDA, that is given by a particular predictor is determined during the OOB error calculation phase whereas the resulting VI value is conditional in the sense of beta coefficients in regression models, but represents the effect of a predictor in both main effects (metabolite-trait-association) and interactions (metabolite-metabolite-interaction) [26].

The mean MDA of each predictor based on the aggregated forest can be used to rank the predictors. In order to reduce the number of metabolites to a manageable size, a permutation test of Hothorn et al. [25] was performed. We set the threshold of the permutation test to p ≤ 0.1 which rejects uninformative predictors and enables the selection of predictor variables with significant importance. Hereby the risk of too many wrongly believed predictive predictors is reduced [27].

The root mean square error (RMSE) of RFR is calculated as the square root of the difference between the realized (*y*_*i*_) and the predicted observation (${\hat{y}}_{i}^{\text{OOB}}$) within the OOB data after permuting each predictor variable in the training dataset divided by the number of trees (*n*).

$${RMSE}_{OOB}\;=\sqrt{\;n^{-1}+\sum_{n=1}^{n}{\left\{y_{i}-\;{\hat{y}}_{i}^{OOB}\right\}}^{2}}$$ (2)

RMSE is calculated at each splitting step in the trees just as averaged over the whole forest. The coefficient of determination (R^2^) of RFR is computed as

$${R^{2}}_{OOB}=\;1-\;\frac{\sum_{i=1}^{n}{\left(y_{i}-{\hat{y}}_{i}^{OOB}\right)}^{2}}{\sum_{i=1}^{n}{\left(y_{i}-\bar{y_{i}}\right)}^{2}}$$ (3)

The enhanced RFR approach of Hothorn et al. [28] is implemented in the R package *party* and its subroutines ‘cforest’ and ‘varimp’ by Strobl et al. [25] were used in this study. All needed settings are realized by the activation of the specifications ‘controls = cforest_unbiased’ in the tree building function ‘cforest’ and ‘conditional = TRUE’ in the VI calculating function ‘varimp’. Because *party* does not provide the OOB error estimation by default, the function ‘postResample’ within R package *caret* was used to calculate RMSE and R^2^ based on the conditional forest learned by ‘cforest’.

RFR calculation, in particular using function ‘varimp’ of *party*, is regarding CPU time and RAM capacity particular in the situation of our large (1993) amount of independent variables very time demanding. To reduce the CPU time of RFR, through a previous selection step, we removed a portion of the apparently uninformative predictors.

Finally RFR was applied on a preselected set of 3 × 400 metabolites, which were most important in the first three PCs according to their absolute loadings. After removing duplicates, 1084 metabolites remained in the final dataset. According to Strobl et al. [26] the number of decision trees (‘ntree’ parameter’) was set to 1084 and the number of candidate predictors at each split (‘mtry’ parameter) was set to 361 (‘ntree’/3). The remaining parameters were set to default.

#### Prediction of response variable using aggregate metabolites profiles

Accuracies in prediction of the meat quality response variables using metabolites profiles were calculated for each applied method via multiple regression analysis. The statistical regression models comprised as independent variables either the first 10 PCs of PCA, 10 modules of WNA or 10 metabolites with highest VI values identified by RFR.

In addition, the results of all analysis were used in a joint analysis in order to identify important biological interpretable networks of metabolites or interactions between promising metabolites and meat quality traits. In this context, the subjective selection of metabolites for the joint analysis based on following conditions: a) metabolite is ranked within the top 30 variables according to their importance indicators (absolute correlation coefficient, absolute loading of PCA, MS of WNA and VI of RFR) in at least one of the applied statistical methods, b) metabolite is annotated. These importance parameters were used to identify metabolites with high meaning for the observed traits. Based on the selected metabolites (six metabolites for each trait) correlation coefficients between metabolites and between metabolites and traits were calculated to construct a network. The software package *pajek* [29] was used to visualize the complex network of all pre-selected metabolites and meat quality traits via arrows and connection lines.

## Results

### Meat quality traits and metabolomic profiling

The raw values of the performance data, given in Table 1, reflect the normal range of meat quality in commercial crossbred pig population. With the exception of the correlation between pH1 and pH24 measured in LD, all correlation coefficients between meat quality parameters were significant different from 0 and had the expected sign (Table 1).

**Table 1:** Descriptive statistics and phenotypic correlations between meat quality traits.

	Mean	S	Min	Max	pH1	pH24	color
drip loss, %	1.90	1.39	0.40	5.30	-0.314**	-0.350***	-0.371***
pH1	6.53	0.22	5.89	6.94		-0.024	0.272**
pH24	5.52	1.12	5.32	6.06			0.638***
color	72.5	7.25	56.00	92.00			

S = standard deviation; Min = minimum; Max = maximum

*** (p ≤ 0.001)

** (p ≤ 0.01); drip loss measured in *Musculus longissimus dorsi* (LD) 24 h post-mortem (p.m.); pH1 measured in LD 45 minutes p.m.; pH24 measured in LD 24 h p.m.; color = meat color measured in LD 24 h p.m.

Untargeted metabolite profiling detected 1993 different metabolites in 97 meat samples. Using Metabolomic Discoveries' databases, 393 metabolites were successfully assigned to a biological function along with a tagged name and description (first choice). In case of 128 annotated metabolites, the *Kyoto Encyclopedia of Genes and Genomes* (KEGG)-IDs were also available. A list of all annotated metabolites is presented in S2 Table. Non-annotated metabolites were characterized by their available exact mass information. In a further step, we tried to annotate the most important metabolites manually by using the METLIN database (second choice). Based on the known accurate mass, neutral charge and a maximal tolerance of +/- 10 ppm a potential functional annotation was assumed for the unknown metabolites.

### Correlation analysis

**Fig 1:**
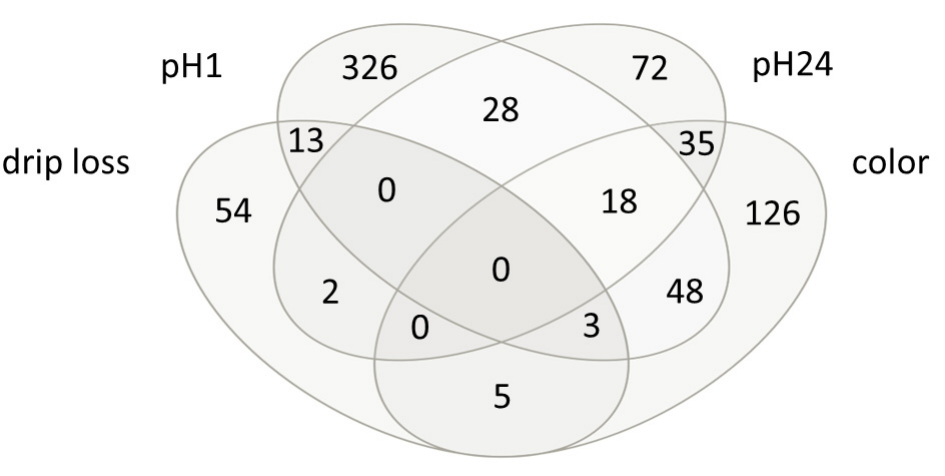
Venn-diagram of significant correlated metabolites. Drip loss measured in *Musculus longissimus dorsi* (LD) 24 h post-mortem (p.m.); pH1 measured in LD 45 minutes p.m.; pH24 measured in LD 24 h p.m.; color = meat color measured in LD 24 h p.m.

The correlation analysis revealed 77, 436, 155 and 235 metabolites significantly correlated with drip loss, pH1, pH24 and color respectively (Table 2). The correlation coefficients ranged from—0.46 to 0.44. As can be seen from the Venn-diagram in Fig 1 most of the relationships were trait specific, whereas only 152 of the 903 significant correlated metabolites showed a significant correlation with more than one meat quality trait. In case of trait meat color more than half of the significant metabolites are also significant correlated with other meat quality trait.

**Table 2:** Results of the correlation analysis for traits and metabolites.

trait	No. positive correlated	range	No. negative correlated	range
drip loss	72	0.20 to 0.25	5	- 0.21 to—0.24
pH1	212	0.20 to 0.44	224	- 0.20 to—0.46
pH24	99	0.20 to 0.41	56	- 0.20 to—0.32
color	162	0.20 to 0.35	73	- 0.20 to—0.37

Drip loss measured in *Musculus longissimus dorsi* (LD) 24 h post-mortem (p.m.); pH1 measured in LD 45 minutes p.m.; pH24 measured in LD 24 h p.m.; color = meat color measured in LD 24 h p.m.; significance threshold p ≤ 0.05.

### Principal component analysis (PCA)

**Fig 2:**
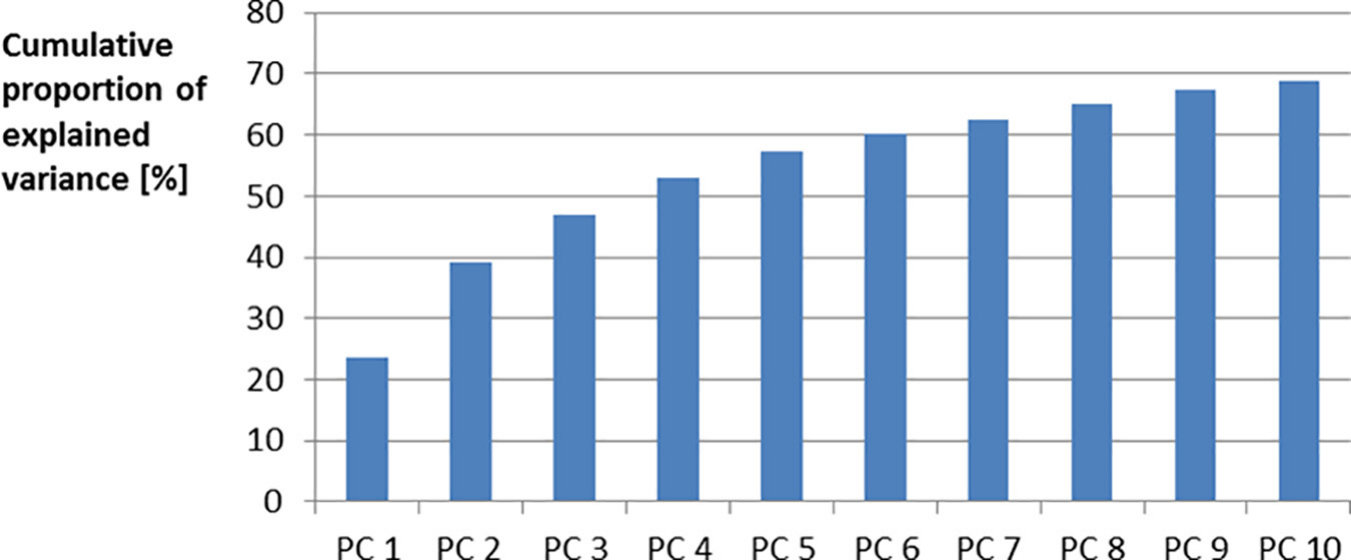
Cumulative proportion of explained variance by principal component one to 10. PC = principal component.

PCA was used to condense expression profiles of all metabolites in a reasonable number of PCs. As shown in Fig 2, the first three PCs already specified 46.9% of the observed variance. These proportion increases with diminishing response of additional PCs from 60% using 6 PC up to 70% using more than 10 PC.

In order to identify significant metabolites, we focused on the first 3 PC as it has been proposed by DiLeo et al. [24]. In these PC the loadings of all metabolites were in a range of -0.1 to + 0.1. According to the criteria to rank loadings in PCs [24], in our study the metabolites do not reach significant eigenvalues of > 0.2 or < - 0.1. Furthermore beneath the possibility to rank the variables, a general biological characterisation of the first PCs is hardly possible.

### Weighted network analysis (WNA)

WNA allowed the entire dataset of 1993 probe sets of metabolites to be utilized in the construction of the weighted co-expression network. The hierarchical clustering algorithm and the following pruning process resulted in 10 modules (Figure A in S1 File). The number of metabolites per module ranged between 776 (module ‘blue’) and 31 (module ‘salmon’). Four metabolites were not assigned to any module, and were labeled with color ‘gray’.

**Fig 3:**
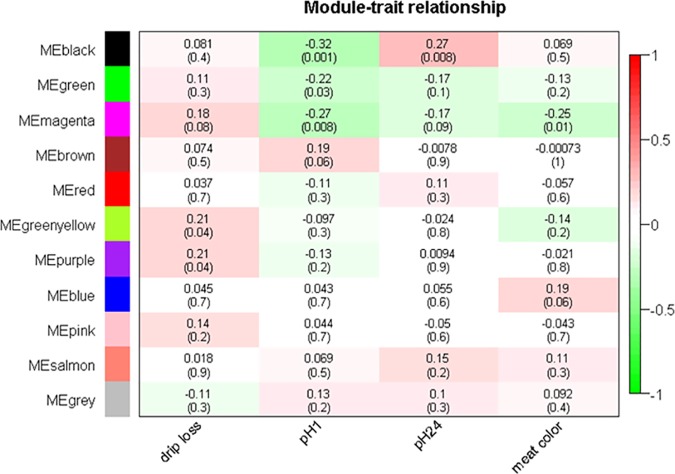
Correlation coefficients and corresponding p-values of module-trait relationship. Correlations of traits drip loss, pH1, pH24 and meat color to modules are characterized by color range from red (‘1’—positive correlation) to green (‘-1’—negative correlation). In parenthesis below correlation coefficients the p-value is given. Drip loss is measured in *Musculus longissimus dorsi* (LD) 24 h post-mortem (p.m.); pH1 measured in LD 45 minutes p.m.; pH24 measured in LD 24 h p.m.; meat color measured in LD 24 h p.m.; ME = module eigenvalues.

The relationships between meat quality traits and modules are given as correlation coefficients between traits and MEs (Fig 3). Drip loss was significant positively correlated with modules ‘purple’ and ‘greenyellow’. Meat color and pH1 showed a significant negative correlation with the module ‘magenta’. MEs of module ‘black’ were significantly correlated to pH24 and pH1, but these coefficients were controversial in sign (Table 3, Fig 3).

**Table 3:** Selection of significant modules for meat quality traits in weighted network analysis.

trait	module	cor.	p-value	number metabolites
drip loss	‚purple‘	+ 0.21	p ≤ 0.04	52
drip loss	‚green-yellow‘	+ 0.21	p ≤ 0.04	49
pH1	‚magenta‘	- 0.27	p ≤ 0.008	53
pH1	‚black‘	- 0.32	p ≤ 0.001	73
ph24	‚black‘	+ 0.28	p ≤ 0.008	73
color	‚magenta‘	- 0.25	p ≤ 0.01	53

cor. = Correlation; drip loss measured in *Musculus longissimus dorsi* (LD) 24 h post-mortem (p.m.); pH1 measured in LD 45 minutes p.m.; pH24 measured in LD 24 h p.m.; color = meat color measured in LD 24 h p.m.

MAR values were calculated using metabolites of the entire data set. However, regarding the MAR calculation of a specific metabolite, it can be expected that the metabolites which belong to the same module provide the most valuable information due to their high intramodular connectivity. The majority of the metabolites had MAR values below 0.2 and only 88 metabolites had MAR values above 0.3.

**Fig 4:**
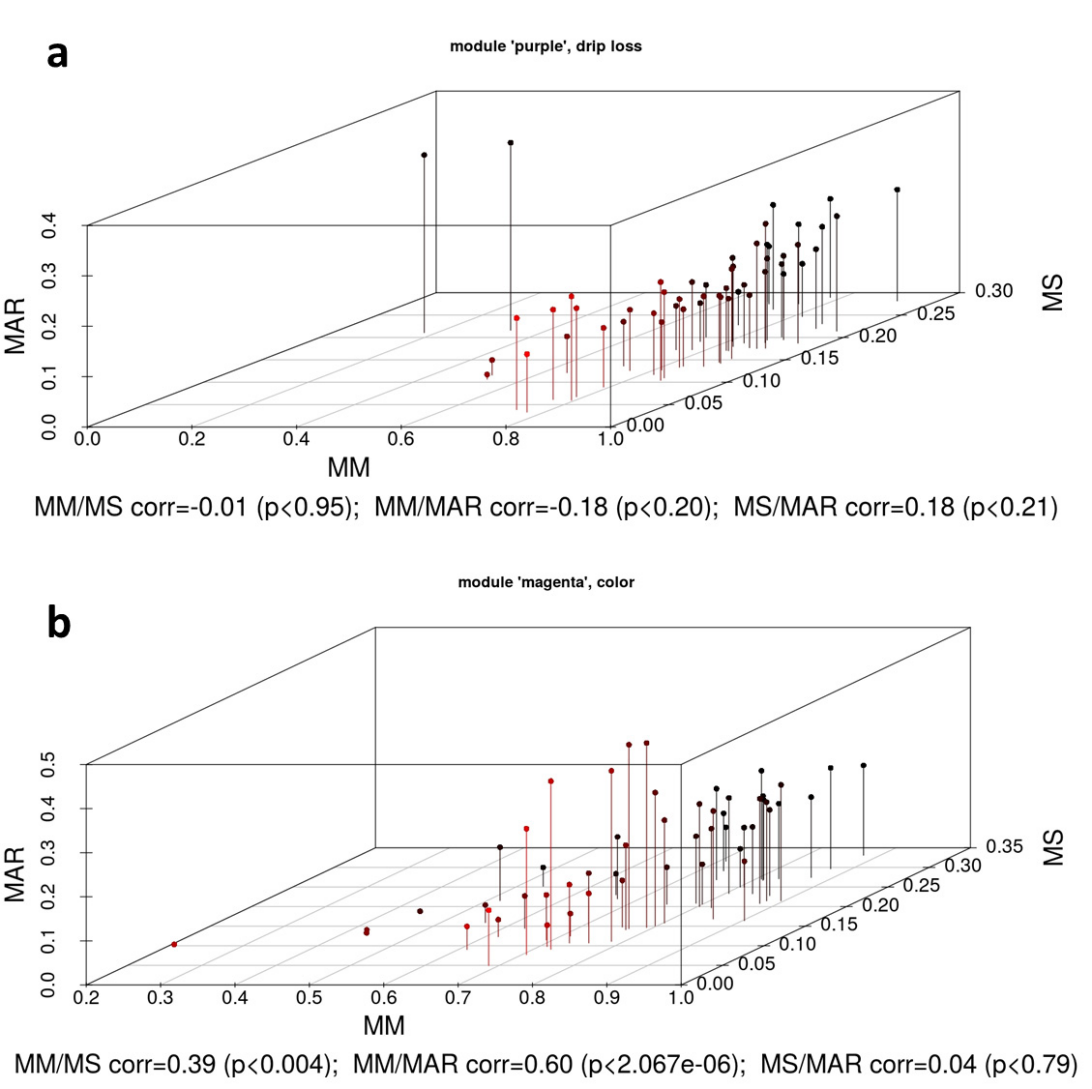
**Scatterplot of parameters metabolite significance, module membership and maximum adjacency ratio of the modules ‘purple’ (a) and ‘magenta’ (b) that are significantly correlated with meat quality traits drip loss (a) and meat color (b).** MS = metabolite significance; MM = module membership; MAR = maximum adjacency ratio; drip loss measured in *Musculus longissimus dorsi* (LD) 24 h post-mortem (p.m.); color = meat color measured in LD 24 h p.m.

Of particular interest were metabolites with high MM, MAR and MS. We used both the ‘not module based’ parameter MAR as well as the ‘module based’ parameters MS and MM to select metabolites that are important from different perspectives. Within the significant modules the metabolite qualifiers MM showed in many cases positive correlation coefficients to MS and MAR estimators (Table 4). For example, in the significant module ‘magenta’ (for trait color) the correlation coefficients MM: MS = 0.39 and MM: MAR = 0.60 indicated the high information content of the MM qualifier not only for the module specific connectivity but also for the response variable and the relativeness of the entire network (Fig 4). Likewise in module ‘black’ (for trait pH1) there were significant positive correlations between MM: MS and MM: MAR. Particular in these modules it can be expected to find a reasonable number of potential key metabolites for meat quality influencing pathways [30].

**Table 4:** Correlation between metabolite significance, module membership and maximum adjacency ratio for modules of weighted network analysis.

	‘purple’, drip loss	‘greenyellow’, drip loss	‘black’, pH1	‘black’, pH24	‘magenta’, pH1	‘magenta’, color
MM: MS	0.00	0.36**	0.55**	-0.13	0.29*	0.39**
MM: MAR	-0.18	0.81**	0.29**	0.29**	0.60**	0.60**
MAR: MS	0.18	0.25	-0.22*	0.50**	-0.14	0.40

MS = metabolite significance; MM = module membership; MAR = maximum adjacency ratio

** (p ≤ 0.01)

* (p ≤ 0.05); drip loss measured in *Musculus longissimus dorsi* (LD) 24 h post-mortem (p.m.); pH1 measured in LD 45 minutes p.m.; pH24 measured in LD 24 h p.m.; color = meat color measured in LD 24 h p.m.

Regarding the relationship MS: MAR a clear tendency were observed only in the modules ‘black’ for trait pH24 and ‘magenta’ for color where the correlation coefficient exceeded a value 0.4. In all other modules this relationships were negative or close to 0. To demonstrate the relationship of MM, MS and MAR, the scatterplots in Fig 4 visualize the relations exemplarily for modules ‘purple’ and ‘magenta’, that were significantly associated with drip loss and meat color. The plots for the remaining module-trait associations can be found in Figure B in S1 File.

### Random forest regression (RFR)

In contrast to the previous approaches, RFR is a supervised learning method characterizing the relationship between trait and metabolites using decision trees. Due to computational problems of 1993 available metabolites only the probably most important 1084 metabolites were used in RFR. These metabolites were selected based on their absolute loading values in PC1 to PC3 in PCA as described above. By this procedure the dataset was reduced from 1993 to 1084 metabolites. Regarding the different meat quality traits, diverse conditional RFR accuracy parameters (RMSE, R^2^ and coefficient of variation (CV)) of the prediction based on metabolite profiles were estimated. R^2^ values ranged between 0.4 (pH24) and 0.55 (pH1). CV values for pH1 (2.21%) and color (6.95%) were below 10%, whereas CV values for pH24 (17.57%) and particular drip loss (51.15%) indicated a weak accuracy of RFR for these traits (Table 5).

**Table 5:** Accuracy parameters and number of metabolites with significant variable importance (VI) and maximal VI per trait according to random forest regression.

trait	RMSE	R^2^	CV [%]	Max. VI	significant metabolites
drip loss	0.97	0.41	51.15	0.012	293
pH1	0.14	0.55	2.21	0.002	401
pH24	0.97	0.40	17.57	0.013	317
color	5.04	0.47	6.95	1.658	332

RMSE—root mean square error; R^2^—coefficient of determination; CV—coefficient of variation; drip loss measured in *Musculus longissimus dorsi* (LD) 24 h post-mortem (p.m.); pH1 measured in LD 45 minutes p.m.; pH24 measured in LD 24 h p.m.; color = meat color measured in LD 24 h p.m.

**Fig 5:**
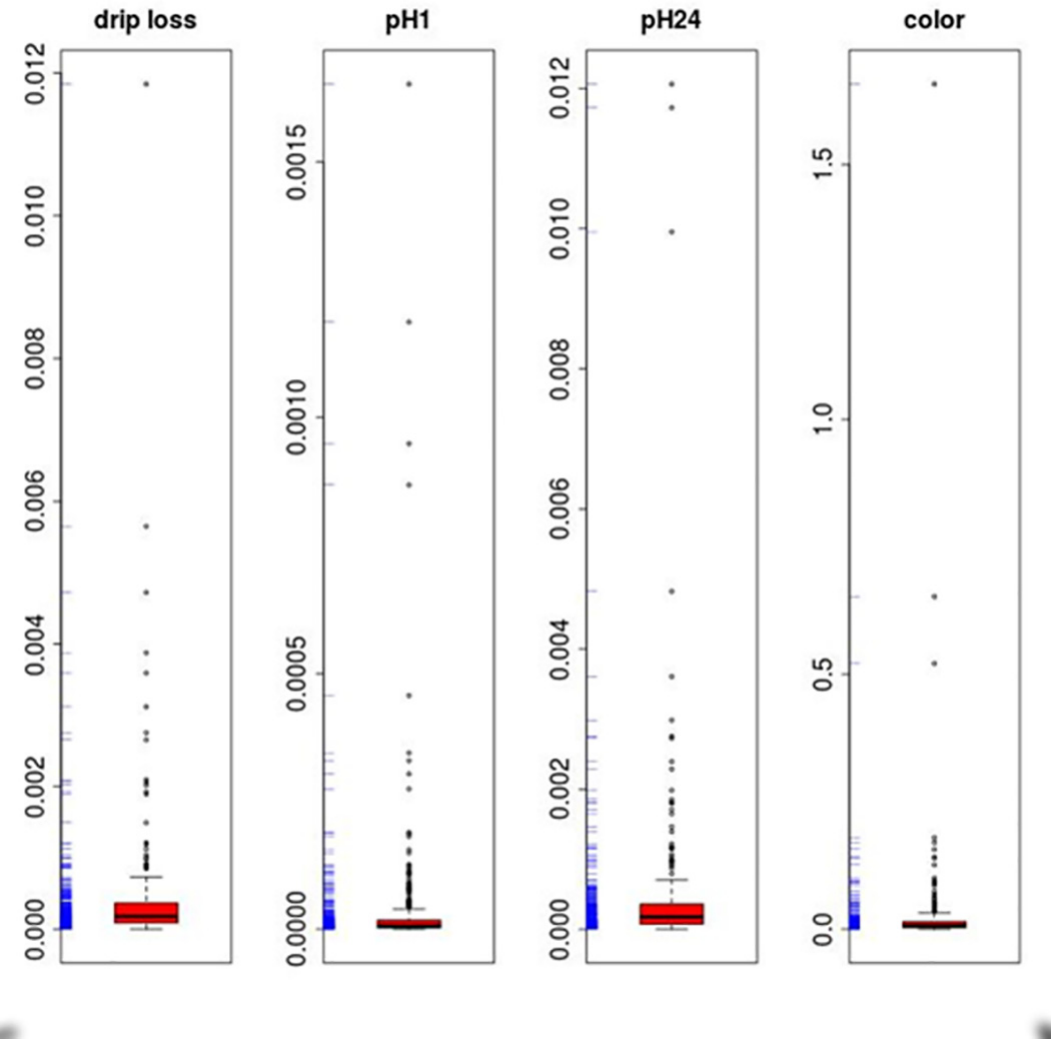
Variable importance boxplot of important metabolites by random forest regression of Strobl et al. (2009) [29]. Drip loss measured in *Musculus longissimus dorsi* (LD) 24 h post-mortem (p.m.); pH1 measured in LD 45 minutes p.m.; pH24 measured in LD 24 h p.m.; color = meat color measured in LD 24 h p.m.

**Fig 6:**
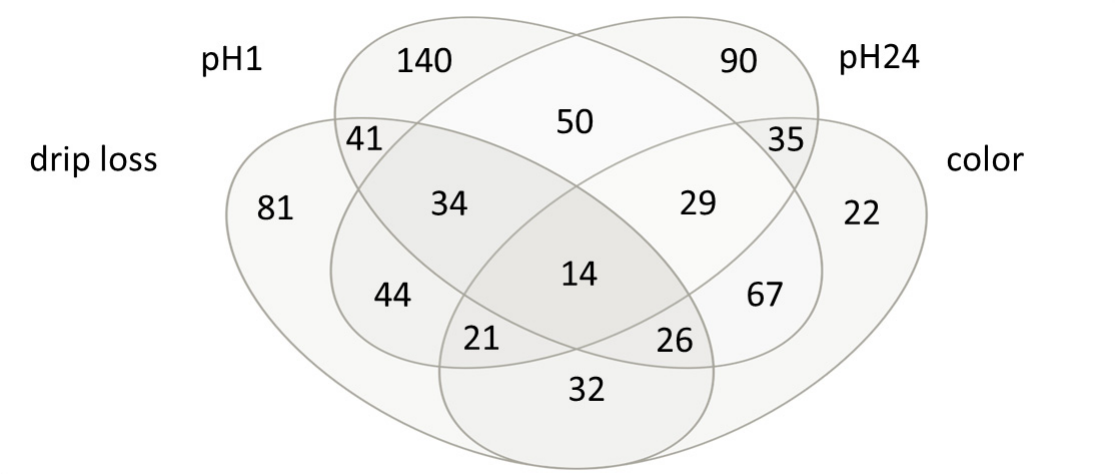
Venn-diagram of significant metabolites by random forest regression of Strobl et al. (2009) [29]. Drip loss measured in *Musculus longissimus dorsi* (LD) 24 h post-mortem (p.m.); pH1 measured in LD 45 minutes p.m.; pH24 measured in LD 24 h p.m.; color = meat color measured in LD 24 h p.m.

Despite these partly unsatisfying accuracies, a considerable number (293 to 401) of metabolites with significant impact on various meat quality traits (Table 5, Fig 5) were detected. Significance of VI values was tested via a permutation test with a threshold of p ≤ 0.1. As shown in Fig 6, there is a large number of metabolites identified for more than one trait. For example, 14 and 110 (34 + 29 + 26 + 21) metabolites had a significant impact on all or at least on three meat quality traits.

### Evaluation of applied statistical methods in prediction of meat quality traits

In a final step, the potential of all applied statistical models to predict meat quality was quantified via a trait specific multiple regression analysis. Regarding the statistical procedures PCA, WNA and RFR the first 10 PCs, all WNA modules or 10 highest RFR VI values were used as independent variables. Table 6 shows the corresponding accuracy parameters of these analyses. In general, prediction based on metabolite profiles was very challenging in case of drip loss and worked best for pH1. Regarding the statistical methods in most analyses RFR showed the highest accuracy. Only for pH24, the first ten PCs and the modules of WNA resulted in higher R^2^ compared to RFR (Table 6).

**Table 6:** Predictive power of principal component analysis, weighted network analysis and random forest regression in drip loss, pH1, pH24 and meat color based on a multiple regression model.

trait	multiple correlation coefficients								
	10 principal components of PCA			10 modules of WNA			10 metabolites with highest variable importance of RFR		
	RMSE	R^2^	CV[%]	RMSE	R^2^	CV[%]	RMSE	R^2^	CV[%]
drip loss	1.13	0.07	59.75	1.10	0.18	57.94	1.10	0.32	58.13
pH1	0.43	0.35	6.53	0.44	0.30	6.69	0.43	0.37	6.64
pH24	0.32	0.27	5.73	0.32	0.27	5.78	0.34	0.12	6.13
color	2.54	0.23	3.50	2.56	0.21	3.53	2.51	0.37	3.46

PCA = principal component analysis; WNA = weighted network analysis; RFR = random forest regression; RMSE—root mean square error; R^2^—coefficient of determination; CV—coefficient of variation; drip loss measured in *Musculus longissimus dorsi* (LD) 24 h post-mortem (p.m.); pH1 measured in LD 45 minutes p.m.; pH24 measured in LD 24 h p.m.; color = meat color measured in LD 24 h p.m.

### Joint analysis of significant associated metabolites

**Fig 7:**
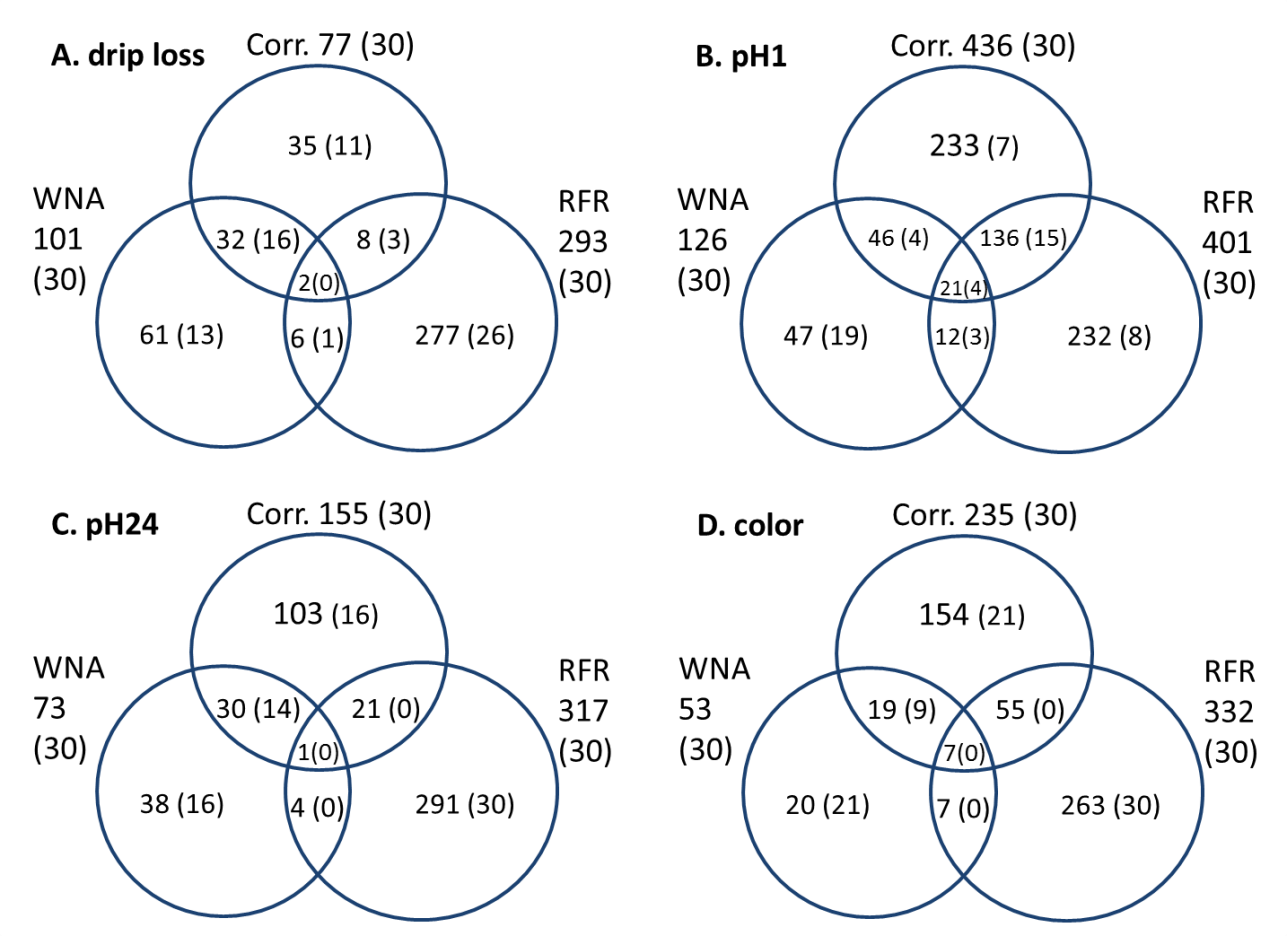
Venn-diagram of the entire significant metabolites for drip loss, pH1, pH24 and color identified by correlation analysis, weighted network analysis and random forest regression and of the selection of 30 metabolites with highest absolute correlation coefficients, metabolite significance and variable importance values in brackets. Corr. = correlation analysis; WNA = weighted network analysis; RFR = random forest regression; drip loss measured in *Musculus longissimus dorsi* (LD) 24 h post-mortem (p.m.); pH1 measured in LD 45 minutes p.m.; pH24 measured in LD 24 h p.m.; color = meat color measured in LD 24 h p.m.

With the exception of PCA, the applied methods revealed significant metabolites for the observed meat quality traits. PCA resulted in weak loading values that prohibited the identification of important metabolites. In contrast, using the results of the correlation analysis and the methods WNA and RFR, it was possible to detect significant associated metabolites for meat quality traits. Comparing these methods by summarizing the results presented in the Tables 2, 3 and 5 the number of detected significant trait specific metabolites varied to a large extent. For example the number of significant metabolites for drip loss ranged from 76 (correlation analysis) to 293 (RFR). On the other hand, a considerable overlapping of significant metabolites identified by different statistical methods was detected and is presented in Fig 7. In general, it can be assumed that metabolites, whose importance is confirmed by different methods, can be used as reliable predictors for meat quality traits.

In order to get a more comprehensive overview about the complex biological architecture of meat quality traits, the most important metabolites that were identified by the three methods were used to set up a network via virtualization tool *pajek*. Importance of metabolites was characterized by the parameters a) correlation to meat quality, b) MS in significant modules of WNA and c) VI in RFR. According to these parameters the most important 30 metabolites per method were preselected. The final joint network analysis comprised only metabolites which were annotated and identified by at least two methods. Following this rule, 6 metabolites were identified for pH1, drip loss and color, whereas 3 metabolites had an impact on pH24. For pH24, this initial subset did not contain results from the RFR analysis, so that the list was extended by three annotated metabolites which had the highest VI value (Table 7).

**Table 7:** Selection of metabolites for joint analysis based on their ranking in top 30 metabolites in correlation analysis, metabolite significance of weighted network analysis and variable importance of random forest regression.

**drip loss**	**Cor**	**MS**	**VI**	**pH1**	**Cor**	**MS**	**VI**
2.3-Naphthalic acid	23.	×	10.	Histidine-alanine-tryptophan-tryptophan	5.	4.	2.
Glycero-3-phosphocholine	8.	×	7.	Cytidine	25.	8.	12.
Glycero-3-phosphoserine	×	28.	23.	Allopurinol-1 ribonucleoside	×	9.	25.
Glycerophosholipid	22.	14.	×	Lactic acid	24.	×	10.
Triacylglycerol	19.	12.	×	Lysine-serine-isoleucine	19.	×	6.
3-Methyl-2-oxovaleric acid	21.	13.	×	Phosphocreatine	26.	×	21.
**pH24**	**Cor**	**MS**	**VI**	**color**	**Cor**	**MS**	**VI**
α-Hydroxybutyrate	1.	1.	×	Octulose-1.8-bisphosphate	7.	1.	×
Heptadecanoyl carnitine	2.	2.	×	Fructose-6-phosphate	27.	9.	×
Stearoylcarnitine	3.	4.	×	Glucose-6-phosphate	23.	7.	×
Gle-cholesterol	×	×	2.	Inosine-5-monophosphate	28.	10.	×
Methylglyoxal	×	×	9.	Phosphoglycolic acid	11.	12.	×
Glucose	×	×	11.	Nicotinamide adenine dinucleotide	4.	×	2.

Cor = correlation analysis; MS = metabolite significance; VI = variable importance; ×—Metabolite was not ranked in top 30 of the respective importance values; drip loss measured in *Musculus longissimus dorsi* (LD) 24 h post-mortem (p.m.); pH1 measured in LD 45 minutes p.m.; pH24 measured in LD 24 h p.m.; color = meat color measured in LD 24 h p.m.; Gle = gallic acid-linoleic acid ester.

**Fig 8:**
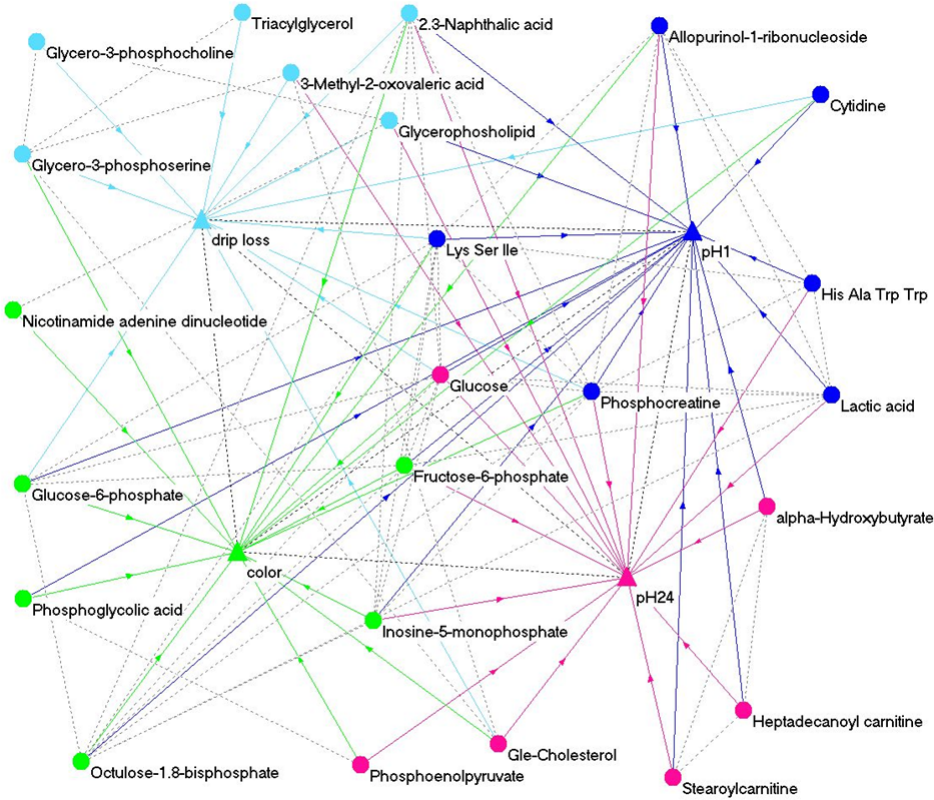
Metabolomic network of traits drip loss, pH1, pH24 and color and 24 strongly associated metabolites. dotted lines: undirected connections between metabolites and between traits; arrows: directed relations between metabolites and traits; triangles—traits; ellipses–metabolites; light blue–drip loss; blue–pH1; magenta–pH24; green–meat color; drip loss measured in *Musculus longissimus dorsi* (LD) 24 h post-mortem (p.m.); pH1 measured in LD 45 minutes p.m.; pH24 measured in LD 24 h p.m.; color = meat color measured in LD 24 h p.m.

Based on the 24 selected metabolites in Table 7, a metabolomic network was created which comprise the meat quality traits drip loss, pH1, pH24 and color (Fig 8). In the network the dotted lines represent connections between traits and between metabolites whereas the arrows stand for directed effects of metabolites on the observed traits. Directed and undirected connections are displayed in case of significance (p ≤ 0.05) and absolute correlation ≥ 0.5. Fig 8 indicates that the metabolites found by different statistical methods were highly interconnected. As a general tendency, different procedures identified similar or related chemical substances for a specific trait.

## Discussion

### Challenges in metabolomics and functional analysis

Based on an untargeted metabolomic approach the main objective of our study was to identify key metabolites which play an important role in the complex biological architecture of meat quality traits. Moreover, these metabolites can be used as informative predictive biomarkers of meat quality of pigs. Similar objectives are pursued in a few studies reported in literature [9, 31]. Recently D'Alessandro et al. [31] successfully used metabolomics to compare highly phenotypically differentiated pig breeds. Rohart et al. [11] investigated the prediction power of metabolomic profiles for commonly used production phenotypes in pig breeds and in a current study Muroya et al. [12] tried to reveal characteristic metabolic pathways in different porcine muscle types. These studies used up to 188 well known metabolites to characterise different targets traits. In contrast to these studies, our approach tried to cover the entire metabolom of pigs expressed in meat samples.

Using a GC-MS technology, in our study 1993 different metabolites were identified. Wishart et al. [32] showed that this method is the most efficient way for metabolite detection. However, as it has been reported by Hollywood et al. [33] metabolomics approaches by GC-MS only disclosure about 10% of the metabolome. From this follows, that the set of metabolites found in our study reflects only a small percentage of anticipated count of metabolites.

Besides this limitation, the corresponding annotation step provides only a fragmented picture because only a small amount of physiological or biochemical functions of metabolites are stored in available public data bases. In our study only 393 out of 1993 metabolites were annotated. According to Chagoyen and Pazos [34], reasons for these fragmentary information might be the of lack of scientific fundamentals and principles of physiological and biochemical processes of higher life forms. In addition, functional analysis of high dimensional omics data is a big challenge in systems biology studies as it can be seen by the different, non-standardized statistical methods which were used here and elsewhere to analyze metabolomics data (cf. [24]).

### Potential and abilities of statistical methods

In order to quantify the consequences of missing statistical standards in a first step of our analysis we evaluated different statistical methods with respect to their relevant theoretical statistical properties and their consequences regarding the final results. All applied methods tried to solve the problem of the ‘large p, small n’ situation of the metabolomics dataset used in our study.

The correlation analysis is a useful method to get a first overview. In the last decade, in many scientific fields we registered an increasing number of available variables. New techniques were proposed to address these challenging tasks involving many irrelevant and redundant variables and often comparably few training examples. Selecting the most relevant variables is a challenge for building a reliable predictor, particularly if the variables are redundant. Conversely, a subset of useful variables may exclude many redundant, but relevant, variables. Correlation methods belong to the category of ranking criteria defined for individual variables, independently of the context of others. This leads to the consequence that some variables may have a low rank because they are redundant and yet be highly relevant [35]. In our study correlation analysis nevertheless induced concordant results with RFR and WNA. The latter finding was not surprising because the module generating process in WNA, MS is calculated based on Pearson correlation coefficients as well as the simple correlation analysis. In conclusion, correlation analysis is a comprehensible procedure to get a first idea of what variables might be potential bio indicators. However, because of the described weaknesses, beneath correlation analysis, other methods based bootstrap or Bayesian procedures should be applied to validate or disprove the first results.

The PCA approach tries to condense the information content of the independent variables into a set of PC. This method is promising, in particular because bivariate correlation analysis revealed significant relationships between metabolites and meat quality traits in a range of maximal—0.20 to—0.46 and + 0.20 to + 0.44. However, using PCA only weak loading values were estimated within the first PC and no significant metabolites were identified based on the thresholds described by DiLeo et al. [24]. Consequently the analytic tool did not give comprehensive insight in interactions between metabolome and phenotypic traits.

WNA addresses the challenge of the ‘large p, small n’ situation by summarizing a network of modules to reduce the complexity of a dataset, which is thereby analyzed with greater statistical power [15]. The investigation and interpretation of trait–metabolite associations in WNA is focused on the most highly-connected ‘hub’ metabolites with high MM, MS and MAR within the significant modules.

As described above, the parameter MS quantifies the importance of a metabolite for meat quality traits. Therefore, MS is the most eligible parameter to select promising metabolite biomarkers for a particular trait. In contrast, the parameters MM or MAR are indicators for the connectivity of metabolites and are able to indicate potential key players in the regulatory network regulating the trait of interest and between the metabolic pathways. MM quantifies the importance of a metabolite for the specific module, whereas the parameter MAR provides information about the relatedness of each metabolite within the whole network. According to Langfelder and Horvath [15] MAR values below 0.5 indicate components with many, weak connections to the network neighbors instead of few strong associations. In our study only four metabolites in the modules ‘black’ and ‘magenta’ showed MAR values above 0.4, which indicated a more important role of these metabolites regarding the network connectivity. In these modules the correlation MS: MAR also revealed a clear tendency of 0.5 (module ‘black’, pH24) and 0.4 (module ‘magenta’, color), respectively (Table 4). In other significant modules the MS: MAR correlations were negligible weak. This finding indicates that an intensively connected metabolite does not necessarily provide important information for the expression of the response variable.

Within significant modules, MM values of almost all metabolites were highly expressed in a range between 0.5 to 1 (Fig 4, Figure B in S1 File). This result can be expected because of the underlying cluster algorithm. In contrast, the MS values within all significant modules were much lower and were almost equally distributed between 0 and 0.3 for drip loss, color and pH1 (in ‘magenta’) and 0 to 0.45 for pH1 (in ‘black’) and pH24 (Table 3). As visualized in Fig 6, in module ‘purple’, glucosylceramide (d18:1/24:1(15Z)) and another unannotated metabolite with low MM but high MAR values do not fit in our expectations derived from a positive correlation between MM and MAR qualifiers. Theoretically, components with low MM but high MAR probably have a high connectivity across the whole network. In this context, glucosylceramide (d18:1/24:1(15Z)) is involved in many pathways for example in sphingolipid-, ceramide glucosyl- and lipopolysaccharide metabolism so that this metabolite can be considered as a nodal point between different modules or metabolic pathways. On the other hand, in module ‘magenta’ there are some metabolites with both high MM and MAR qualifiers, but MS values close to 0 (Fig 4). These metabolites might be key players in the underlying biological pathways of module ‘magenta’, but on the other side they do not play an important role for the expression of the response variable color by its own.

According to Muroya et al. [12] module construction and MM/MS/MAR calculation is reasonable, because it can be expected that the biology of meat ageing process is regulated by a number of key factors in several key metabolic pathways. Module construction has the advantage that the function of a large amount of non-annotated metabolites can be inferred from their better-annotated neighbors within the modules. This advantage is particular important in the analysis of our data set, because only 20% of the metabolites were annotated.

In a PCA the meaning of metabolites is calculated purely by their statistical correlation (covariance) to all other metabolites. This means, that the significance of metabolites with high regulatory importance, but no directly connected to the trait of interest (weak loadings), is not detected by a PCA. In contrast, in the framework of the WNA analysis metabolites which have a central position within a regulatory network have a higher probability to be identified [24]. Varying the minimum number of metabolites within a module has an impact on the module sizes and the total number of modules identified. This option allows the user to consider biological background knowledge. Choosing a lower number of variables per module allows the user to investigate the underlying biological pathways more in detail. In conclusion, these attributes of WNA provided evidence that the procedure was an appropriate method for analyzing metabolomics data in a system biology approach.

As a final result, the WNA procedure leads to differentiable modules with similar expression profiles within the modules. From a biological point of view the intra module similarity can be interpreted as a distinct co-regulation of the module metabolites. Moreover, the indicators MM and MAR enables to identify key players in regulatory network which is possibly linked to the specific module, whereas metabolites strongly influencing specific traits are characterized by high MS [15]. Nowadays weighted co-expression network analyses are applied in a wide scientific field in order to estimate the relationships, connectivity and dependency of different variables in biological systems. In metabolomics approaches the combined abilities of WNA to cluster and select variables are also very useful. For example, DiLeo et al. [24] and MacLennan el al. [36] successfully used WNA to select metabolite biomarkers in tomatoes and transcripts as biomarkers in mice.

In RFR, VI is usually used for selection of (a) causal variables highly related to the response variable for explanatory and interpretation purposes and (b) of a small number of relevant predictor variables. It was shown in test runs using all independent variables (results not shown), many of the metabolites had very little importance in the trees and therefore in prediction of the trait of interest. Despite the expectation that the RFR procedure is able to handle high dimensional data with redundant and unimportant variables, the analysis ran more robust and in acceptable running time based on the reduced dataset with 1084 instead of 1993 metabolites.

To deal with the impurity’s bias for selecting split variables towards uncorrelated predictor variables, VI values were calculated with an enhanced RFR procedure that guarantees unbiased tree algorithms for reliable prediction and interpretability in both individual trees and forests. In standard RFR, by Breiman [14], the VI based ranking of the predictors says nothing about the significance of the top-ranked predictors and the procedure always outputs a ranking–even if all predictors are uninformative in the prediction. In contrast, in Hothorn´s conditional RFR [28], VI computation is based on an implemented permutation test which analyzed the significance of the respective metabolite. This selection step leads to a reduced number of explanatory variables in the model that avoids overfitting and ensures a smaller prediction error [11]. Generally, the VI parameter in RFR can be interpreted similar to MS values in WNA. In contrast to WNA, which determines MM of each metabolite within a module, RFR does not estimate the relative similarity among metabolites. This limitation of the RFR procedure makes it difficult to assign metabolites to different functional pathways. Moreover, RFR approaches partially produce ‘odd unexpected results’ in some specific cases [37]. Even in the enhanced conditional RFR procedure, the risk of biased VI values in case of specific data structure or predictor type cannot be overlooked completely. As well as the pretended advantage of RFR, the absence of a specific underlying stochastic model, is also a challenge in the sense that it is difficult to understand how the prediction within the variety of decision trees works exactly [37]. Nevertheless, RFR has become a major analysis tool in many fields of bioinformatics due to its high flexibility and in-build VI calculation. Also in prediction of various characteristics based on metabolomics data, RFR has been used successfully [38].

### Accuracy of prediction of meat quality traits by applied methods

To evaluate the prediction ability of meat quality traits by PCA, WNA and RFR a linear (multiple) regression model was used. Suitability of the methods in prediction on basis of metabolite profiles was different regarding the different traits (Table 6). Compared to drip loss, pH1 and color prediction performed better using the 10 selected metabolites by RFR, whereas pH24 prediction based on PCs or WNA modules resulted in higher R^2^. According to the studies of Rohart et al. [11], who also used RFR for phenotypic prediction based on metabolomic data, prediction accuracy depends strongly on the observed trait. In our study, prediction worked best for the trait pH1 and worst for drip loss, considering R^2^. This result might correspond to the genetic foundation of these traits. It has been summarized by Ciobanu et al. [39] that the lowest heritability estimates were found for drip loss whereas pH1, pH24 and color showed higher values (h^2^ drip loss = 0.31, h^2^ pH1 = 0.41, h^2^ pH24 = 0.39, h^2^ color = 0.57). That means, drip loss is stronger influenced by environmental effects which might complicate the prediction accuracy by metabolite information.

### Joint analysis

A network of metabolites and meat quality traits is represented in Fig 8. Trait pH1 was the most cross-linked trait in our study and several metabolites like 2,3-naphthalic acid and glucose were significantly associated with all respected traits. Moreover glucose, selected due to high importance for trait meat color, was connected to eight other metabolites, amongst others to inosine-5-monophosphate (IMP), lactic acid and 2,3-naphtalic acid. Besides its influence on meat color, the metabolite IMP that is involved in purine metabolism and biosynthesis of alkaloids derived from histidine and purine, also showed significant associations to drip loss, pH1 and pH24. Taking into account the significant phenotypic correlation among the four traits as well (Table 1), all observations indicated that meat quality traits were highly interconnected and influenced by similar biochemical processes.

Regarding the different statistical approaches it can be summarized that the applied procedures all in all identified similar or related chemical substances as important for a specific meat quality trait. For example in regard to of drip loss, correlation analysis, WNA and RFR revealed several glycerophospholipids (GPL) and glycerolipids (GL) that are involved in lipid metabolism and arise from degradation of membrane structures. Moreover, similar to the findings of Hidalgo et al. [40], different acids, like 2,3-naphthalic acid and the α-keto acid 3-methyl-2-oxovaleric acid, that are associated with lipid oxidation were identified by different methods as important for drip loss. For example 2,3-naphthalic acid is part of the pathway ‘degradation of aromatic compounds’ that directly leads to generation of pyruvic acid and other compounds that are involved in energy metabolism like acetic acids. Most important metabolic processes in muscle and meat are energetic processes like glycolysis/gluconeogenesis, citrate cycle and pentose phosphate pathway (PPP), which verifiable are responsible for muscle physiology and meat quality [41, 42].

In hypoxic tissues after slaughtering anaerobe metabolic processes predominate and in glycolysis glycogen is released via glucose to pyruvic acid. Under aerobic conditions pyruvic acid is metabolized in citrate cycle and oxidative phosphorylation [42]. In case of stress before slaughtering or a too short resting period before stupefaction in hypoxic tissues, the rate of oxidative processes like glycolysis is increased and pyruvic acid do not flow into glycolysis but is transferred to lactic acid [17]. Accumulation of lactic acid goes along with pH decrease to 5.6 [17]. The coincidence of low pH1 and high temperature in muscle lead to partial denaturation of proteins and reduction of intercellular space. Thereby, lipids are dissolved from membranes, permeability of membranes is increased and drip loss is the result [3]. Based on this background, the meaning of e.g. 2,3-naphthalic acid, glucose and several GPLs, sterol lipids and fatty acids for meat quality characteristics drip loss, pH1, pH24 and color is traceable. These metabolites are indicators for complex metabolic processes and are characteristic of the specific occurrence of meat quality traits. Selected metabolites potentially may be used as universal bio indicators for prediction of special traits. Availability of such ‘multiple applicable’ biomarkers would reduce effort and cost of phenotyping in breeding programs and commercial meat processing. Regarding the associations between the metabolites, it was observed that some metabolites were significantly correlated with many other components. This finding suggested that some strongly networked metabolites are the key players of metabolic processes responsible for the large complex of meat quality traits in pigs. Intense investigation of these important metabolites might lead to a deeper understanding of the underlying biological pathways and the causal reasons of development of quality traits.

### Key metabolites and apparently significant associated pathways related to meat quality traits

The different applied methods resulted in several key metabolites mainly belonging to the family of lipids (GPLs, sterol lipids, prenol lipids). In addition to lipids, the statistical analysis also detected other compounds like the naphthalene 2,3-Naphthalic acid and the α-keto acid 3-methyl-2-oxovaleric acid with strong association to drip loss. GPLs are the major lipids in mammalian cell membranes [43]. Preslaughter stress results in increased rate and extent of pH decline, decomposition of membrane structures and cell swelling and shrinkage. In this way dissolved lipids and lipid compounds run off the cells into the extramyofibrillar compartment. This process of lipid decomposition also is accompanied by lipid oxidation that results in increasing concentration α-keto acids. Therefore the relation between drip loss and associated lipids and acids can be explained and have been already described by Lambert et al. [43] and Poulsen et al. [44].

Examination of compounds with significant association to pH1 resulted in metabolites of purine and pyrimidine metabolism (nucleotide metabolism), glycolysis and PPP. PH1 is a major indicator for PSE (pale, soft, exudative) meat, which is characterized by low pH1. The higher the rate of glycolysis, PPP and related metabolic processes like lactic acid—and nucleotide metabolism, the lower is pH1 in meat. The onset of rigor mortis at low pH1 and high temperature causes the denaturation of around 20% of the sarcoplasmic and myofibrillar proteins [17]. This explains the significant meaning of polypeptides like histidine-alanine-tryptophan-tryptophan and lysine-serine-isoleucine.

Trait pH24 was significant associated with metabolites of pyruvate metabolism, glycolysis, PPP and purine metabolism. Moreover, pH24 was significant associated to metabolites resulting in the course of protein degradation (e.g. polypeptide glutamine-histidine-alanine) and metabolites of lipid metabolism, like GPs, sterol lipids and fatty acid esters (e.g. stearoylcarnitine), and hydroxy acids like α-hydroxybutyrate (ketone body). The meat quality parameter pH24 is an indicator for DFD (dark, firm, dry) meat, which characteristically leads to a pH ultimate value > 6. High ultimate pH results in relative little protein degradation, high WHC, dark meat and early spoilage of the meat. Meat spoilage follows from microbial reduction, natural autoxidation of lipids and autolytic enzymatic processes [17]. Toldra and Flores [45] reported the significance of fatty acids and ketones and polypeptides (products of autolytic enzymatic spoilage) for pH24 in meat. The degradation of free fatty acids to ketone bodies in liver is one option to generate energy for muscle cells. With empty glycogen stores p.m. energy is mainly supplied by mobilization of lipid stores and transformation of released fatty acids into ketone bodies [17]. Because these anaerobic processes lead to reduced pH decline p.m. several fatty acids and ketone bodies (e.g. α-hydroxybutyrate) might be good indicators for pH24. Relevance of p.m. energy metabolisms like glycolysis, PPP and pyruvic acid metabolism, indicated by metabolites like glucose and phosphoenolpyruvate, for meat quality traits in pigs also has been described by Scheffler et al. [42].

Analysis of pork color resulted in different significant associated metabolites (phosphates, pyruvic acid) of glycolysis, PPP and pyruvic acid metabolism. This means high rate of glycolysis and activated PPP and pyruvate metabolism results in high meat color value (Opto value, scattering effect), because glucose is metabolized to glycogen and finally to lactic acid. This goes along with acidification and pale meat color and explains the determined significant meaning of phosphates and downstream products of glycolysis like octulose-1,8-bisphosphate and phosphoglycerate in our study. Muroya et al. [12] and D'Alessandro et al. [31] who investigated characteristic metabolic pathways of meat quality in pigs could confirm these results. They also indicated significant correlation coefficients between meat color indicators (L*, a*, b*) and higher rate of glycolysis.

## Conclusion

In this study untargeted metabolite profiling of muscle samples of 97 Duroc × Pietrain pigs was used to identify underlying biochemical processes and potential key molecules affecting meat quality traits. Because of limited technical capabilities of GC-MS and a lack of basic knowledge about biochemical processes of higher life forms only detection and annotation of a small percentage of metabolites influencing meat quality was possible. To get deeper insights in the involved biological pathways we applied and evaluated different statistical methods, namely correlation analysis, PCA, WNA and RFR. Although the methods based on different statistical approaches and in spite of differences between the parameters and requirements of the particular methods to achieve statistical significance, they revealed similar results. Using the described methods for analysis of the holistic metabolite profiling we were able to detect both: metabolites with already known meaning for meat quality and metabolites whose influence on meat quality traits not yet has been described. As expected, the applied methods revealed metabolites as important, that are involved in p.m. glycogen degradation and energy consumption under the exclusion of oxygen like glucose, GPLs and different phosphates. On the other hand the meaning of several metabolites like e.g. the polypeptides histidine-alanine-tryptophan-tryptophan and lysine-serine-isoleucine for trait pH1 has not yet been described in literature.

The consistent results lead to the conclusion that meat quality traits pH1, pH24 and color are strongly influenced by processes of p.m. energy metabolism like glycolysis, PPP, pyruvic acid metabolism and associated processes. Drip loss in particular is significant associated with different glycerophospho-, sterol- and prenol lipids and compounds involved in lipid metabolism which are products of membrane degradation. In summary, it was possible to attain findings on the interaction of meat quality traits and their underlying biochemical processes. The detected key molecules will be used in further investigations in order to clarify the complex molecular structures underlying drip loss. Furthermore, these selected metabolites might be better indicators of meat quality especially of drip loss than the measured phenotype itself and potentially might be used as bio indicators. For this purpose the validation of the candidate bio indicators in another set of pigs is desirable.

## Supporting Information

S1 File **Text A. The random forest regression procedure of Breiman can be subdivided into a series of 6 steps; Text B. Differences between the traditional random forest regression of Breiman and conditional inference forests; Figure A. Module identification in weighted network analysis based on a cluster dendrogram and merging of co-regulated modules; Figure B. Scatterplot of parameters metabolite significance, module membership and maximum adjacency ratio of the modules ‘greenyellow’ (a), ‘black’ (b, d) and ‘magenta’ (c) that are significantly correlated with meat quality traits drip loss (a), pH1 (b, c) and pH24 (d)**.
(PDF)

S1 Table Raw data of meat quality parameters and all quantified metabolites. (ZIP)

S2 Table List of annotated metabolites. (CSV)

## References

[1] Verbeke W, Perez-Cueto F. J. A., de Barcellos M. D., Krystallis A, Grunert KG. European citizen and consumer attitudes and preferences regarding beef and pork. Meat Sci. 2010; 84: 284–292. 10.1016/j.meatsci.2009.05.001 20374787

[2] Schafer A, Rosenvold K, Purslow PP, Andersen HJ, Henckel P. Physiological and structural events postmortem of importance for drip loss in pork. Meat Sci. 2002; 61: 355–366. 10.1016/S0309-1740(01)00205-4 22061063

[3] Huff-Lonergan E, Lonergan SM. Mechanisms of water-holding capacity of meat. The role of postmortem biochemical and structural changes. Meat Sci. 2005; 71: 194–204. 10.1016/j.meatsci.2005.04.022 22064064

[4] Liu G, Jennen D. G. J., Tholen E, Juengst H, Kleinwachter T, Holker M, et al A genome scan reveals QTL for growth, fatness, leanness and meat quality in a Duroc-Pietrain resource population. Anim Genet. 2007; 38: 241–252. 10.1111/j.1365-2052.2007.01592.x 17459017

[5] Ponsuksili S, Jonas E, Murani E, Phatsara C, Srikanchai T, Walz C, et al Trait correlated expression combined with expression QTL analysis reveals biological pathways and candidate genes affecting water holding capacity of muscle. BMC Genomics. 2008; 9 10.1186/1471-2164-9-367 PMC2529315 18671879

[6] Terenina E, Babigumira BM, Le Mignon G, Bazovkina D, Rousseau S, Salin F, et al Association study of molecular polymorphisms in candidate genes related to stress responses with production and meat quality traits in pigs. Domest Anim Endocrin. 2013; 44: 81–97. 10.1016/j.domaniend.2012.09.004 23063408

[7] Te Pas M. F. W., Hoekman A. J. W., Smits MA. Biomarkers as management tools for industries in the pork production chain. Journal on Chain and Network Science. 2011; 10: 155–166.

[8] Liu JS, Damon M, Guitton N, Guisle I, Ecolan P, Vincent A, et al Differentially-expressed genes in pig longissimus muscles with contrasting levels offFat, as identified by combined transcriptomic, reverse transcription PCR, and proteomic analyses. J Agr Food Chem. 2009; 57: 3808–3817. 10.1021/Jf8033144 19296579

[9] Bertram HC, Oksbjerg N, Young JF. NMR-based metabonomics reveals relationship between pre-slaughter exercise stress, the plasma metabolite profile at time of slaughter, and water-holding capacity in pigs. Meat Sci. 2010; 84: 108–113. 10.1016/j.meatsci.2009.08.031 20374761

[10] Te Pas M, Kruijt L, Smits M. Use of biomarkers as tools for tracking and tracing meat and meat products and to predict and monitor meat quality. In: Maltin C, Craigie C, Bünger L, editors; 2012.

[11] Rohart F, Paris A, Laurent B, Canlet C, Molina J, Mercat MJ, et al Phenotypic prediction based on metabolomic data for growing pigs from three main European breeds. J Anim Sci. 2012; 90: 4729–4740. 10.2527/jas.2012-5338 23100586

[12] Muroya S, Oe M, Nakajima I, Ojima K, Chikuni K. CE-TOF MS-based metabolomic profiling revealed characteristic metabolic pathways in postmortem porcine fast and slow type muscles. Meat Sci. 2014; 98: 726–735. 10.1016/j.meatsci.2014.07.018 25105492

[13] Gromping U. Variable Importance Assessment in Regression. Linear regression versus random forest. Am Stat. 2009; 63: 308–319. 10.1198/tast.2009.08199

[14] Breiman L. Random forests. Mach Learn. 2001; 45: 5–32. 10.1023/A:1010933404324

[15] Langfelder P, Horvath S. WGCNA. an R package for weighted correlation network analysis. BMC Bioinformatics. 2008; 9 10.1186/1471-2105-9-559 PMC2631488 19114008

[16] Zentralverband der Deutschen Schweineproduktion, ZDS. Richtlinien für die Stationsprüfung auf Mastleistung, Schlachtkörperwert und Fleischbeschaffenheit beim Schwein. Bonn; 2003.

[17] Honikel KO, Kim CJ. Causes of the development of PSE pork. Fleischwirtschaft. 1986; 66: 349–353.

[18] Lisec J, Schauer N, Kopka J, Willmitzer L, Fernie AR. Gas chromatography mass spectrometry-based metabolite profiling in plants. Nat Protoc. 2006; 1: 387–396. 10.1038/nprot.2006.59 17406261

[19] Jolliffe IT, Trendafilov NT, Uddin M. A modified principal component technique based on the LASSO. J Comput Graph Stat. 2003; 12: 531–547. 10.1198/1061860032148

[20] Fuller TF, Ghazalpour A, Aten JE, Drake TA, Lusis AJ, Horvath S. Weighted gene coexpression network analysis strategies applied to mouse weight. Mamm Genome. 2007; 18: 463–472. 10.1007/s00335-007-9043-3 17668265 PMC1998880

[21] Dong J, Horvath S. Understanding network concepts in modules. BMC Syst Biol. 2007; 1 10.1186/1752-0509-1-24 PMC3238286 17547772

[22] Horvath S, Dong J. Geometric interpretation of gene coexpression network analysis. Plos Comput Biol. 2008; 4 10.1371/journal.pcbi.1000117 PMC2446438 18704157

[23] Horvath S. Weighted network analysis. Applications in genomics and systems biology New York: Springer; 2011.

[24] DiLeo MV, Strahan GD, den Bakker M, Hoekenga OA. Weighted correlation network analysis (WGCNA) applied to the tomato fruit metabolome. Plos One. 2011; 6 10.1371/journal.pone.0026683 PMC3198806 22039529

[25] Strobl C, Boulesteix AL, Zeileis A, Hothorn T. Bias in random forest variable importance measures. Illustrations, sources and a solution. BMC Bioinformatics. 2007; 8 10.1186/1471-2105-8-25 PMC1796903 17254353

[26] Strobl C, Boulesteix AL, Kneib T, Augustin T, Zeileis A. Conditional variable importance for random forests. BMC Bioinformatics. 2008; 9 10.1186/1471-2105-9-307 PMC2491635 18620558

[27] Nicodemus KK, Malley JD, Strobl C, Ziegler A. The behaviour of random forest permutation-based variable importance measures under predictor correlation. BMC Bioinformatics. 2010; 11 10.1186/1471-2105-11-110 PMC2848005 20187966

[28] Hothorn T, Hornik K, Zeileis A. Unbiased recursive partitioning: A conditional inference framework. J Comput Graph Stat. 2006; 15: 651–674. 10.1198/106186006X133933

[29] Batagelj V, Mrvar A. Pajek—Analysis and visualization of large networks. Lect Notes Comput Sc. 2002; 2265: 477–478.

[30] Oldham MC, Horvath S, Geschwind DH. Conservation and evolution of gene coexpression networks in human and chimpanzee brains. Proc Natl Acad Sci U S A. 2006; 103: 17973–17978. 10.1073/pnas.0605938103 17101986 PMC1693857

[31] D'Alessandro A, Marrocco C, Zolla V, D'Andrea M, Zolla L. Meat quality of the longissimus lumborum muscle of Casertana and Large White pigs. Metabolomics and proteomics intertwined. J Proteomics. 2011; 75: 610–627. 10.1016/j.jprot.2011.08.024 21920477

[32] Wishart DS. Advances in metabolite identification. Bioanalysis. 2011; 3: 1769–1782. 10.4155/Bio.11.155 21827274

[33] Hollywood K, Brison DR, Goodacre R. Metabolomics. Current technologies and future trends. Proteomics. 2006; 6: 4716–4723. 10.1002/pmic.200600106 16888765

[34] Chagoyen M, Pazos F. Tools for the functional interpretation of metabolomic experiments. Brief Bioinform. 2013; 14: 737–744. 10.1093/bib/bbs055 23063930

[35] Guyon I, Nikravesh M, Gunn S, Zadeh LA. Feature Extraction Foundations and Applications. Berlin, Heidelberg: Springer Berlin Heidelberg; 2006.

[36] MacLennan NK, Dong J, Aten JE, Horvath S, Rahib L, Ornelas L, et al Weighted gene co-expression network analysis identifies biomarkers in glycerol kinase deficient mice. Mol Genet Metab. 2009; 98: 203–214. 10.1016/j.ymgme.2009.05.004 19546021

[37] Boulesteix A, Janitza S, Kruppa J, Koenig IR. Overview of random forest methodology and practical guidance with emphasis on computational biology and bioinformatics. Wiley Interdiscip Rev Data Min Knowl Discov. 2012; 2: 493–507. 10.1002/widm.1072

[38] Acharjee A, Kloosterman B, de Vos, R. C. H., Werij JS, Bachem C. W. B., Visser R. G. F., et al Data integration and network reconstruction with similar to omics data using Random Forest regression in potato. Anal Chim Acta. 2011; 705: 56–63. 10.1016/j.aca.2011.03.050 21962348

[39] Ciobanu DC, Lonergan SM, Huff-Lonergan EJ. Genetics of Meat Quality and Carcass Traits In: Rothschild MF, Ruvinsky A, editors. The Genetics of the Pig: CAB International; 2011 p. 358.

[40] Hidalgo FJ, Navarro JL, Delgado RM, Zamora R. Determination of alpha-keto acids in pork meat and Iberian ham via tandem mass spectrometry. Food Chem. 2013; 140: 183–188. 10.1016/j.foodchem.2013.02.052 23578631

[41] Binke R. From muscle to meat. Fleischwirtschaft. 2004; 84: 224–227.

[42] Scheffler TL, Gerrard DE. Mechanisms controlling pork quality development. The biochemistry controlling postmortem energy metabolism. Meat Sci. 2007; 77: 7–16. 10.1016/j.meatsci.2007.04.024 22061391

[43] Lambert IH, Nielsen JH, Andersen HJ, Ortenblad N. Cellular model for induction of drip loss in meat. J Agr Food Chem. 2001; 49: 4876–4883. 10.1021/Jf010121y 11600038

[44] Poulsen KA, Young JF, Theil P, Kolko M, Oksbjerg N, Lambert IH. Role of phospholipase A(2) in the induction of drip loss in porcine muscle. J Agr Food Chem. 2007; 55: 1970–1976. 10.1021/Jf062341n 17288434

[45] Toldra F, Flores M. The use of muscle enzymes as predictors of pork meat quality. Food Chem. 2000; 69: 387–395. 10.1016/S0308-8146(00)00052-2

