# Supplementary material for: Different Statistical Approaches to Investigate Porcine Muscle Metabolome Profiles to Highlight New Biomarkers for Pork Quality Assessment
Source: PLoS One. 2016 Feb 26;11(2):e0149758. doi: 10.1371/journal.pone.0149758 (PMC4769069; doi:10.1371/journal.pone.0149758)
Supplement: S1 File — Text A. The random forest regression procedure of Breiman can be subdivided into a series of 6 steps; Text B. Differences between the traditional random forest regression of Breiman and conditional inference forests; Figure A. Module identification in weighted network analysis based on a cluster dendrogram and merging of co-regulated modules; Figure B. Scatterplot of parameters metabolite significance, module membership and maximum adjacency ratio of the modules ‘greenyellow’ (a), ‘black’ (b, d) and ‘magenta’ (c) that are significantly correlated with meat quality traits drip loss (a), pH1 (b, c) and pH24 (d). (PDF) [file pone.0149758.s001.pdf]

Supporting Information 1 File

Text A. The random forest regression procedure of Breiman can be subdivided into a series of 6 steps.

1. Sampling randomly selected subsets of meat quality observations and subsets of metabolite profiles via bootstrapping. Two-thirds of the data goes into the train data to construct the tree; One-third of the data (‘Out-of-bag’ (OOB) data) is used to estimate the OOB error of the grown tree (validation strep)
2. At each split randomly selecting a subset of predictors (‘mtry’) from the train data.
3. Growing a single regression trees by recursively splitting the subset of metabolites in the subset of predictors. At each node, split the data using the best predictor out of the subset of ‘mtry’ predictors. Tree construction is stopped when tree growth stopping criteria are fulfilled.
4. Estimating the OOB error by applying the tree to the OOB data. The resulting parameters ‘coefficient of determination’ (R^2) and ‘root mean square error’ (RMSE) express the suitability of the tree for prediction of meat quality in independent samples.
5. Generating a random forest as collection of trees by repeating the steps 1-4 ‘ntree’ times.
6. Aggregation of the trees and, based on the entire forest, measurement of the final variable importance (VI) values of the metabolites. Additionally, R^2 and RMSE are averaged over the forest and represent the final parameters of prediction accuracy.

Text B. Differences between the traditional random forest regression of Breiman and conditional inference forests.

In essence, random forest regression (RFR) algorithm of Breiman [1] and Hothorn et al. [2] differs with respect to the a) splitting criteria, b) the resampling scheme and c) the way the predictions of each tree are aggregated to produce a coincident prediction.

a) Splitting criteria: In contrast to Breiman´s RFR [1], the conditional RFR according to Hothorn et al. [2] uses the 'conditional inference forest' (CIF) methodology as splitting criterion. At each splitting node, each predictor is globally tested for its association with the trait of interest and a p-value is computed. Hence, CIF splitting is based on an essentially unbiased splitting criterion that automatically adjusts for different marginal distributions of the predictors and thus does not share the pitfall of Breiman´s RFR.
b) Resampling scheme: The resampling scheme in conditional RFR based on subsampling instead of bootstrap sampling and Strobl et al. [3] recommend to systematically use sampling without replacement to prevent biases in VI measurement.
c) Aggregation procedure: In the conditional RFR it works by averaging the observation weights extracted from each of the trees and not by averaging predictions directly (majority voting).

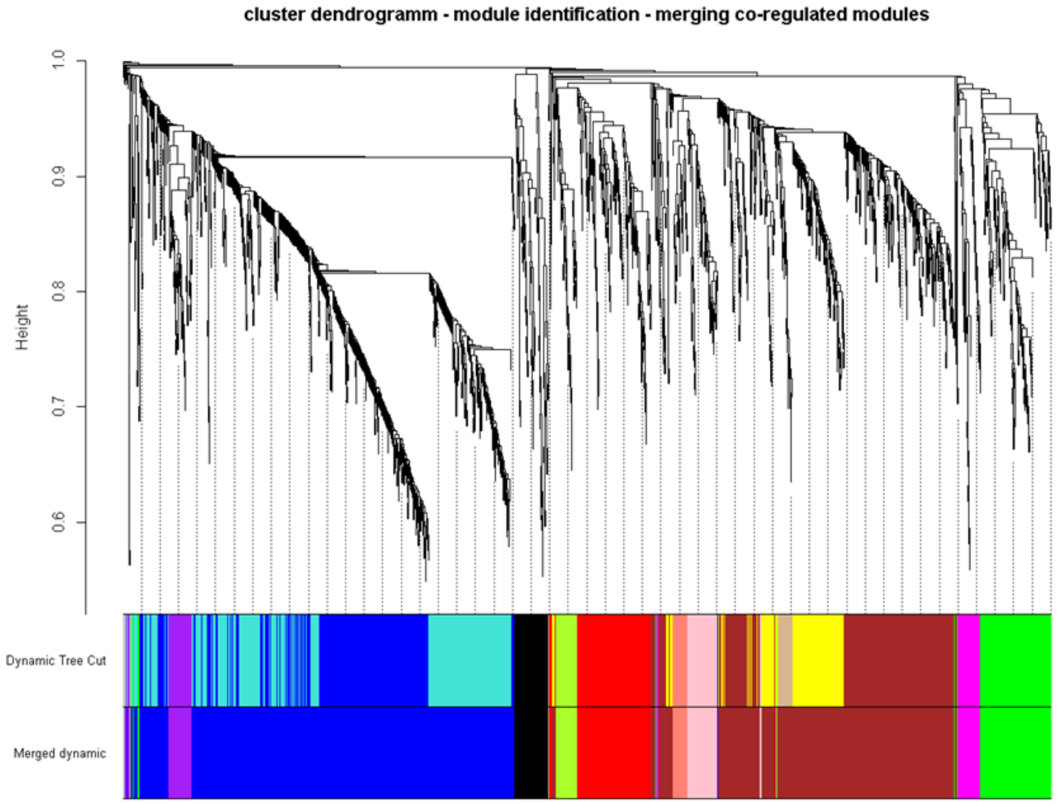

Figure A. Module identification in weighted network analysis based on a cluster dendrogram and merging of co-regulated modules.

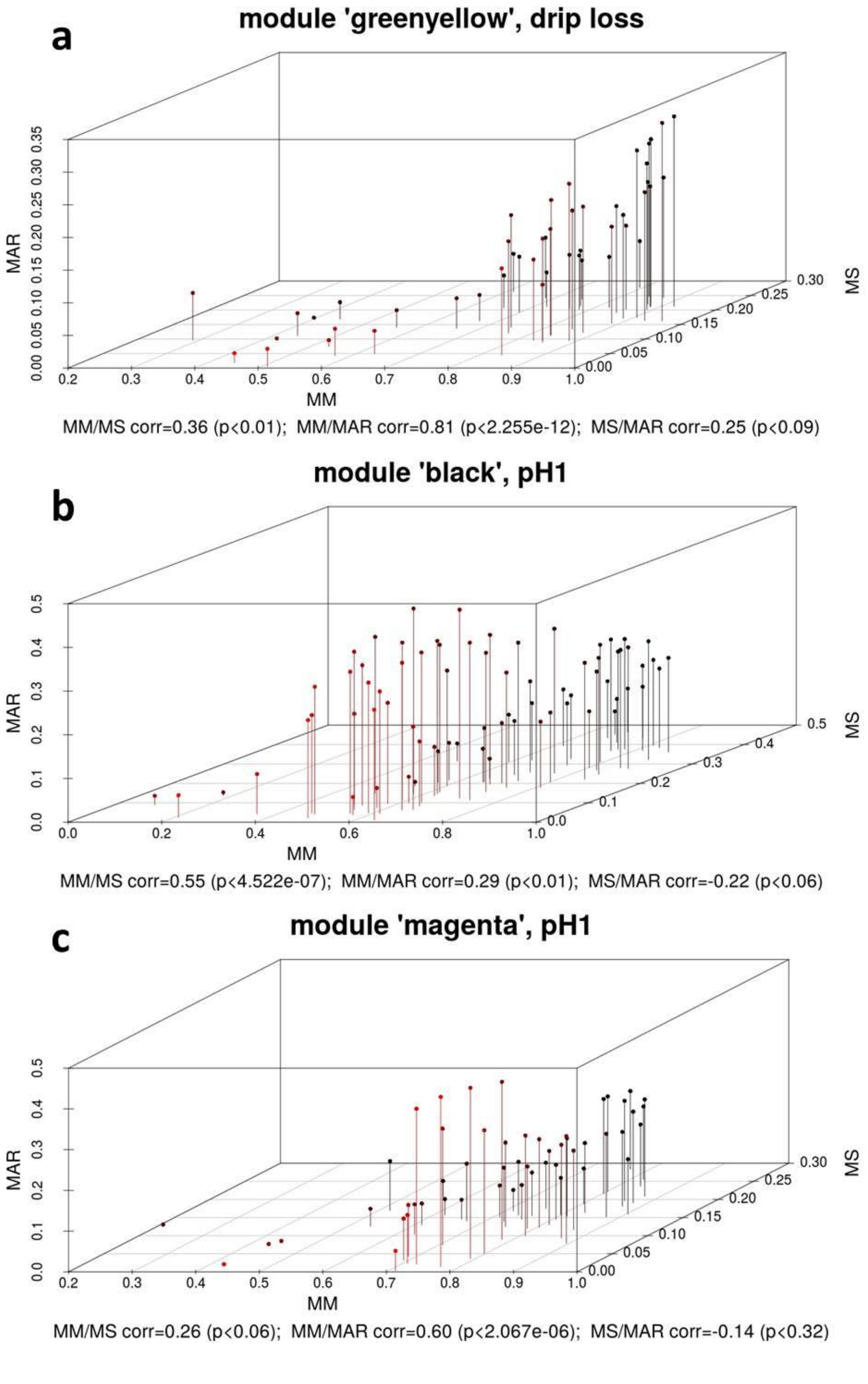
a
module 'greenyellow', drip loss
MAR
MM
MS
MM/MS corr=0.36 (p<0.01); MM/MAR corr=0.81 (p<2.255e-12); MS/MAR corr=0.25 (p<0.09)
b
module 'black', pH1
MAR
MM
MS
MM/MS corr=0.55 (p<4.522e-07); MM/MAR corr=0.29 (p<0.01); MS/MAR corr=-0.22 (p<0.06)
c
module 'magenta', pH1
MAR
MM
MS
MM/MS corr=0.26 (p<0.06); MM/MAR corr=0.60 (p<2.067e-06); MS/MAR corr=-0.14 (p<0.32)

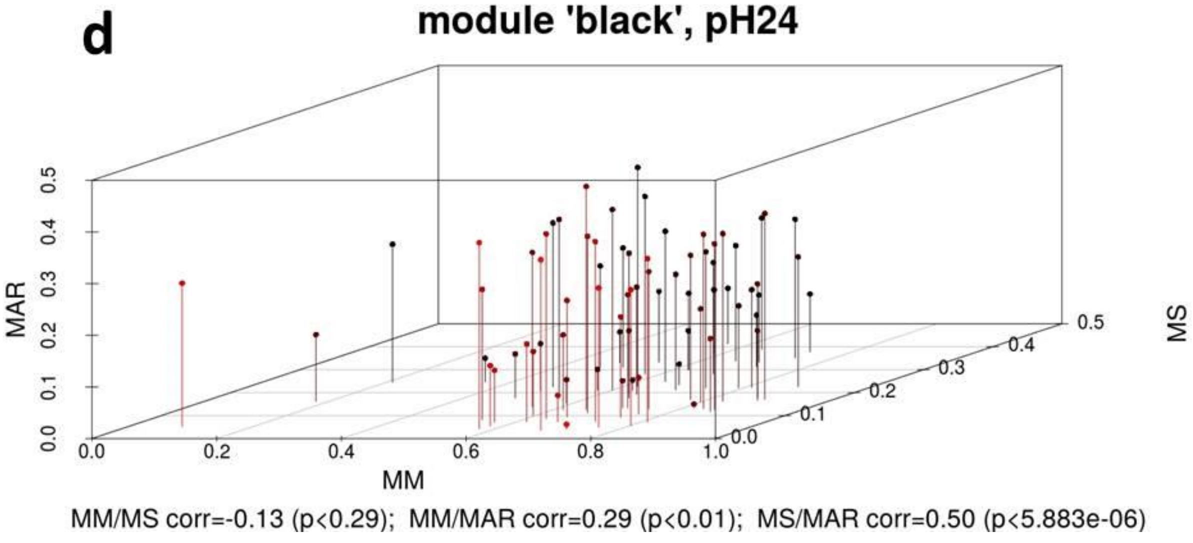

Figure B. Scatterplot of parameters metabolite significance, module membership and maximum adjacency ratio of the modules ‘greenyellow’ (a), ‘black’ (b, d) and ‘magenta’ (c) that are significantly correlated with meat quality traits drip loss (a), pH1 (b, c) and pH24 (d). Metabolite significance = MS; module membership = MM; maximum adjacency ratio = MAR; corr = Pearson correlation coefficient; drip loss measured in *Musculus longissimus dorsi* (LD) 24 h post-mortem (p.m.); pH1 measured in LD 45 minutes p.m.; pH24 measured in LD 24 h p.m.

References

1. Breiman L. Random forests. Mach Learn. 2001; 45: 5–32. doi: 10.1023/A:1010933404324.
2. Hothorn T, Hornik K, Zeileis A. Unbiased recursive partitioning: A conditional inference framework. J Comput Graph Stat. 2006; 15: 651–674. doi: 10.1198/106186006X133933.
3. Strobl C, Boulesteix AL, Kneib T, Augustin T, Zeileis A. Conditional variable importance for random forests. BMC Bioinformatics. 2008; 9. doi: 10.1186/1471-2105-9-307.
